# Heterotrophic Foraminifera Capable of Inorganic Nitrogen Assimilation

**DOI:** 10.3389/fmicb.2020.604979

**Published:** 2020-12-03

**Authors:** Clare Bird, Charlotte LeKieffre, Thierry Jauffrais, Anders Meibom, Emmanuelle Geslin, Helena L. Filipsson, Olivier Maire, Ann D. Russell, Jennifer S. Fehrenbacher

**Affiliations:** ^1^Biological and Environmental Sciences, Faculty of Natural Sciences, University of Stirling, Stirling, United Kingdom; ^2^School of GeoSciences, Grant Institute, University of Edinburgh, Edinburgh, United Kingdom; ^3^Laboratory for Biological Geochemistry, School of Architecture, Civil and Environmental Engineering (ENAC), Ecole Polytechnique Fédérale de Lausanne (EPFL), Lausanne, Switzerland; ^4^UMR CNRS 6112 LPG, Bio-Indicateurs Actuels et Fossiles, Université d’Angers, Angers, France; ^5^Ifremer, IRD, Univ Nouvelle–Calédonie, Univ La Réunion, CNRS, UMR 9220 ENTROPIE, Nouméa, New Caledonia; ^6^Centre for Advanced Surface Analysis, Institute of Earth Sciences, University of Lausanne, Lausanne, Switzerland; ^7^Department of Geology, Lund University, Lund, Sweden; ^8^Université de Bordeaux, EPOC, UMR 5805, Talence, France; ^9^CNRS, EPOC, UMR 5805, Talence, France; ^10^Department of Earth and Planetary Sciences, University of California, Davis, Davis, CA, United States; ^11^College of Earth, Ocean, and Atmospheric Sciences, Oregon State University, Corvallis, OR, United States

**Keywords:** nitrogen cycle, heterotrophic protists, foraminifera, ammonium assimilation, heterotrophy, marine

## Abstract

Nitrogen availability often limits biological productivity in marine systems, where inorganic nitrogen, such as ammonium is assimilated into the food web by bacteria and photoautotrophic eukaryotes. Recently, ammonium assimilation was observed in kleptoplast-containing protists of the phylum foraminifera, possibly via the glutamine synthetase/glutamate synthase (GS/GOGAT) assimilation pathway imported with the kleptoplasts. However, it is not known if the ubiquitous and diverse heterotrophic protists have an innate ability for ammonium assimilation. Using stable isotope incubations (^15^N-ammonium and ^13^C-bicarbonate) and combining transmission electron microscopy (TEM) with quantitative nanoscale secondary ion mass spectrometry (NanoSIMS) imaging, we investigated the uptake and assimilation of dissolved inorganic ammonium by two heterotrophic foraminifera; a non-kleptoplastic benthic species, *Ammonia* sp., and a planktonic species, *Globigerina bulloides*. These species are heterotrophic and not capable of photosynthesis. Accordingly, they did not assimilate ^13^C-bicarbonate. However, both species assimilated dissolved ^15^N-ammonium and incorporated it into organelles of direct importance for ontogenetic growth and development of the cell. These observations demonstrate that at least some heterotrophic protists have an innate cellular mechanism for inorganic ammonium assimilation, highlighting a newly discovered pathway for dissolved inorganic nitrogen (DIN) assimilation within the marine microbial loop.

## Introduction

The nitrogen (N) cycle is one of the most complex marine biogeochemical cycles, and N exerts significant influence on the cycles of many other elements, particularly carbon and phosphorous, because it is often among the elements that can limit biological productivity (Gruber, 2008). In today’s ocean, all the major reactions in the N-cycle are mediated by biology, whether they are assimilatory processes generating organic matter (nitrate/nitrite/ammonium assimilation, N_2_-fixation), or dissimilatory processes generating energy (nitrification, denitrification, and anammox) (Gruber, 2008). Microalgae, cyanobacteria, bacteria, and archaea are the key drivers of N-cycle transformations. At the same time, pelagic and benthic consumers, including heterotrophic protists, contribute to the dissolved organic nitrogen pool through grazing, cell lysis and leakage, and to ammonification, transforming organic nitrogen to ammonium (Mulholland and Lomas, 2008; Kuypers et al., 2018).

Benthic foraminifera are heterotrophic protists that inhabit the marine sediments ranging from salt marshes and intertidal zones to the deep-sea trenches (Murray, 2006), and planktonic foraminifera occupy all open ocean surface waters, occasionally down to 4,000 m (Schiebel and Hemleben, 2017). Their diets vary, but include phytodetritus, bacteria, algae, phyto- and zooplankton, and nematodes (Moodley et al., 2000; Pascal et al., 2008; Dupuy et al., 2010; Enge et al., 2016; Bird et al., 2017, 2018; Chronopoulou et al., 2019). In addition, some species are mixotrophic (Mitra et al., 2016); they house algal symbionts (Gastrich, 1987; Spero, 1987) or kleptoplasts (Jauffrais et al., 2018) that provide fixed carbon to the host whilst also maintaining a heterotrophic mode of feeding (Jauffrais et al., 2016; LeKieffre et al., 2018b).

Foraminifera are highly significant global calcifiers, buffering ocean carbonate chemistry and contributing up to 60% of the total deep-marine calcite budget (Schiebel, 2002; Schiebel et al., 2007). The geochemistry of their fossilized shells is used as a proxy to reconstruct past seawater temperature, pH and other environmental parameters that provide essential constraints for refining climate change projections (Katz et al., 2010). In addition, N-isotopic ratios of the intra-shell proteins are a potential proxy for the supply of nitrate in oligotrophic environments over time (Smart et al., 2018). The last 15 years however, has seen an increase in biological studies revealing that that foraminifera are significant mediators in the N-cycle. Many species can take up and store large intracellular pools of nitrate (Piña-Ochoa et al., 2010) to perform complete denitrification (Risgaard-Petersen et al., 2006; Woehle et al., 2018). Estimates of their contribution to denitrification in marine sediments range from 8% to over 90% (Høgslund et al., 2008; Piña-Ochoa et al., 2010) and hence these abundant ubiquitous microorganisms are of high significance to the global nitrogen budget (Glock et al., 2013). In fact, some benthic foraminiferal species, from the Peruvian oxygen minimum zone, are not just facultative anaerobes switching to nitrate respiration when oxygen is depleted, denitrification is their preferred respiratory pathway (Glock et al., 2019).

In contrast to the ability of some benthic foraminifera to store nitrate for dissimilatory purposes, two kleptoplastic benthic foraminifera (the intertidal *Haynesina germanica* and the subtidal aphotic zone dweller *Non-ionellina labradorica*) assimilate ammonium into their cells (LeKieffre et al., 2018b; Jauffrais et al., 2019). However, in these two studies it could not be verified whether the ammonium assimilation took place through a kleptoplastic pathway (GS/GOGAT enzymatic pathway), or through a foraminiferal pathway; either via a putative mitochondrial glutamate dehydrogenase (GDH) pathway or an alternative GS/GOGAT pathway (van Heeswijk et al., 2013; LeKieffre et al., 2018b; Jauffrais et al., 2019). Thus, the question remains as to whether heterotrophic protists can assimilate ammonium themselves.

Here we demonstrate the ability of two species of heterotrophic foraminifera, the benthic *Ammonia* sp., and the planktonic *Globigerina bulloides*, to take up and assimilate ammonium for cell growth. The genus *Ammonia* is ubiquitous in the sediments of intertidal zones of tropical to temperate waters (Murray, 2006). *Ammonia* sp. can ingest full diatoms into its cytoplasm, and observations of chloroplasts and diatoms in degradation have been reported (LeKieffre et al., 2017). However, *Ammonia* sp. is not able to maintain ingested diatom chloroplasts for more than 24 h and is not a kleptoplastic species (Jauffrais et al., 2016). *G. bulloides* is planktonic and barren of algal symbionts, and is often used in experiments for comparison with symbiont-harboring planktonic species (e.g., Hönisch et al., 2003). Its lack of symbionts also simplifies interpretation of downcore geochemical records based on fossil tests. This species is found across subpolar, temperate and subtropical oceans, and lower latitude upwelling regions (Darling and Wade, 2008), where it frequently dominates the flux of foraminiferal shells to the sea floor. *G. bulloides* has therefore become of considerable importance for palaeoclimate reconstructions (Kleijne et al., 1989; Sautter and Thunell, 1991; Naidu and Malmgren, 1996; Spero and Lea, 1996).

We investigated the uptake of two isotopically labeled micronutrients, ^13^C-bicarbonate (NaH^13^CO_3_) and ^15^N-ammonium (^15^NH_4_Cl), the former to confirm the heterotrophic nature of the investigated specimens, and the latter to investigate the potential for heterotrophic eukaryotic cell uptake of inorganic N.

## Materials and Methods

### Benthic Sampling and Incubation With H^13^CO_3_^–^, and ^15^NH_4_^+^

*Ammonia* sp. specimens were taken from an intertidal mudflat in Fiskebäckskil Harbor, Gullmar Fjord, Skagerrak (West coast of Sweden; 58.24 N, 11.46 E). Previous sampling indicates that the *Ammonia* genotype present in this location is *Ammonia* T6 (Holzmann and Pawlowski, 2000). Therefore, it is probable that the *Ammonia* sp. specimens collected for this study are also T6, but since multiple genotypes can exist in a single location (e.g., Bird et al., 2020) and genotyping cannot be carried out on samples for TEM analysis we have elected to call our specimens *Ammonia* sp.

Living foraminifera were collected in May 2016, at low tide. Only the top 5 mm of the sediment were sampled and immediately transported in the dark to the laboratory. The incubation was carried out at the Kristineberg Marine Research Station, University of Gothenburg (Sweden) the day after collection. In the laboratory, the sediment was sieved on a 200 μm mesh with natural surface seawater directly pumped from the fjord. Only the fraction >200 μm was used. Living individuals of *Ammonia* sp. were selected under a binocular microscope based on cytoplasm color (yellow-brownish material spread through all but the last chamber of the specimen) the day before the experiment and left overnight at 10°C in a Petri-dish filled with artificial seawater to allow them to digest any plastid from algal prey that could interfere with the experiment (Jauffrais et al., 2016). The sampling information for each specimen is described in Supplementary Table S1.

The next day the cytoplasm was checked again, and 10 living specimens were selected and placed into 2 new plastic Petri-dishes (5 specimens per Petri dish) for the experiment. One Petri dish was filled with artificial seawater (ASW, Red Sea Salt, salinity = 34) spiked with 2 mM NaH^13^CO_3_ and 10 μM ^15^NH_4_Cl (Cambridge isotope Inc., Tewksbury MA, United States). The other Petri dish was filled with un-spiked ASW (Red Sea Salt, salinity = 34). The specimens incubated with un-spiked ASW were used as controls for NanoSIMS analysis; see below. The incubation was carried out in a cold room at 10°C with a light source set at 90 μmol photon m^–2^ s^–1^ to mimic their natural environment. After 20 h of incubation with constant light, the foraminifera were immediately chemically fixed by transferring them individually to 0.5 mL microtubes filled with fixative solution for onward TEM-NanoSIMS studies.

### Planktonic Sampling and Incubation With H^13^CO_3_^–^ and ^15^NH_4_^+^

Plankton specimens were collected approximately 1–2 km from Santa Catalina Island, Southern California Bight (33.473 N, 118.485 W). The oceanographic setting near Santa Catalina is fully described by Bird et al. (2017). The seasonal variation in foraminiferal abundances and species composition is well-documented (Thunell and Sautter, 1992; Field, 2004). Specimens for this study were collected in Aug/Sept 2015, during a very strong El Niño event that followed a period of prolonged high sea surface temperature over the preceding 2 years (Bond et al., 2015). This caused an unusual foraminiferal assemblage, more typical of tropical, oligotrophic waters, resulting in low numbers of the ordinarily abundant symbiont barren heterotrophic species, *G. bulloides*. Therefore, a second species of planktonic foraminifera (*Orbulina universa*), was collected at the same time and location as *G. bulloides.* This second species was used as an isotopically normal control for NanoSIMS analysis (Supplementary Table S1). δ^13^C and δ^15^N natural isotopic bulk biomass values of the species *G. bulloides* and *O. universa* sampled were measured from the same location by Uhle et al. (1997). These values were shown to vary within 5 per mil difference from one species to another, which is below the measured variations of our control values (±2σ) (Supplementary Table S2). Indeed, NanoSIMS imaging, while resolving a great spatial resolution, does not allow the resolution of natural isotopic variations.

Samples were collected by scuba diving or net tows. Tow material was transferred to a container filled with ambient surface seawater and kept chilled during transit to shore at the Wrigley Marine Science Centre, where live foraminifera were wet picked. Using binocular light microscopy, individual *G. bulloides* and *O. universa* specimens were identified to the morphospecies level and transferred to 0.2 μm filter-sterilized seawater (FSW) in 20 ml culture jars and for recovery over 2 days. Culture jars were kept in a seawater bath at the ambient temperature of the surface waters throughout the recovery and experiment. Recovery was measured by the regeneration of spines and active feeding by the specimen on pre-frozen *Artemis* nauplii. Only recovered individuals were used in the experiment. The sampling information for each specimen is described in Supplementary Table S1.

For heavy isotope exposure, recovered specimens were gently transferred with a wide glass pipette into new individual 20 ml glass culture jars, filled completely with 0.2 μm FSW spiked with 2 mM NaH^13^CO_3_ and 10 μM ^15^NH_4_Cl (Cambridge Isotopes Inc.). The capped vials were immersed in a water bath held at 22°C under artificial light (Sylvania F24T12 Cool White fluorescent lights). Control specimens (*O. universa)* were treated in the same way without exposure to the heavy isotope spike. *G. bulloides* specimens were incubated with the spike for 6 h (*n* = 2) or 18 h (*n* = 2) before fixing for TEM. Specimens were chemically fixed by transferring individuals immediately to microtubes filled with fixative solution for onward TEM-NanoSIMS studies.

### Preparation for TEM-NanoSIMS Studies

Specimens were fixed in 4% glutaraldehyde and 2% paraformaldehyde diluted in cacodylate (NaCaco) buffer (0.1 M NaCaco, 0.4 M sucrose, and 0.1 M NaCl, pH = 7.4; *Ammonia* sp.) or in 0.8 μm FSW (*G. bulloides*, *O. universa*) at room temperature for 24 h. Fixed cells were stored at 4°C until further processing. The chemically fixed *Ammonia* sp. and *G. bulloides* specimens were decalcified, embedded in resin and sectioned for transmission electron microscopy (TEM) observations as described by LeKieffre et al. (2018a, c). Thin-sections were imaged with a transmission electron microscope, either a Philips 301 CM100 at the Electron Microscopy Facility of the University of Lausanne (Switzerland) or a JEOL JEM-1400 Plus TEM at the Electron Microscopy Facility of the University of Edinburgh (United Kingdom). The integrity of the mitochondria and the membranes of all specimens were checked by TEM observations to ensure the vitality of the studied specimens.

### Stable Isotope Mapping With NanoSIMS

NanoSIMS analytical procedures followed those described by LeKieffre et al. (2018b, c). Areas of interest for NanoSIMS imaging were selected from TEM images. NanoSIMS imaging was carried out on a Cameca 50L NanoSIMS ion microprobe. Images were obtained by bombarding thin sections with a beam of Cs + focused to a spot size of ∼120 nm (beam current ∼2 pA) and counting ^12^C^12^C^–^, ^13^C^12^C^–^-, ^12^C^14^N^–^, and ^12^C^15^N^–^ (see Supplementary Figure S1), in electron multipliers at a mass resolution of about 8,000, enough to resolve potential interferences in the mass spectrum. Each NanoSIMS image consisted of six to eight sequential images that were drift corrected and accumulated using the software L’IMAGE (developed by Dr. Larry Nittler, Carnegie Institution of Washington, United States). The quantified ^13^C/^12^C and ^15^N/^14^N ratios were obtained by the ratio of ^12^C^13^C^–^ with ^12^C^12^C^–^ and of ^12^C^15^N^–^ with ^12^C^14^N^–^, respectively, as follows:

$$\delta^{13}C\left(\%\right)=\left(\left(C_{\mathrm{mes}}/C_{\mathrm{nat}}\right)-1\right)\times 10^{3}$$

$$\delta^{15}N\left(\%\right)=\left(\left(N_{\mathrm{mes}}/N_{\mathrm{nat}}\right)-1\right)\times 10^{3}$$

where C_*mes*_ and N_*mes*_ are the ^13^C^12^C/^12^C_2_ and ^12^C^15^N/^12^C ^14^N ratios measured in the samples and C_*nat*_ and N_*nat*_ are the same ratios measured in an isotopically normal control sample (i.e., the *Ammonia* sp. and *O. universa* controls).

For *G. bulloides* and *Ammonia* sp. regions of interest (ROIs) were drawn with the software L’IMAGE to quantify mean ^15^N enrichments and standard deviations of different sub-cellular structures of a given foraminifera (Supplementary Table S2). Note that TEM-NanoSIMS sample preparation leads to the loss of all soluble compounds. Therefore, only ^13^C and ^15^N assimilated in the biomass can be measured (Nomaki et al., 2018; Gibbin et al., 2020; Loussert-Fonta et al., 2020). *T*-tests were performed to verify whether there was a significant difference in the δ^15^N and δ^13^C of different cellular compartments of *Ammonia* sp. (cytoplasm, electron opaque bodies and nucleoli) compared with the cytoplasm control values (Supplementary Table S2).

## Results

### TEM Observations

**FIGURE 1:**
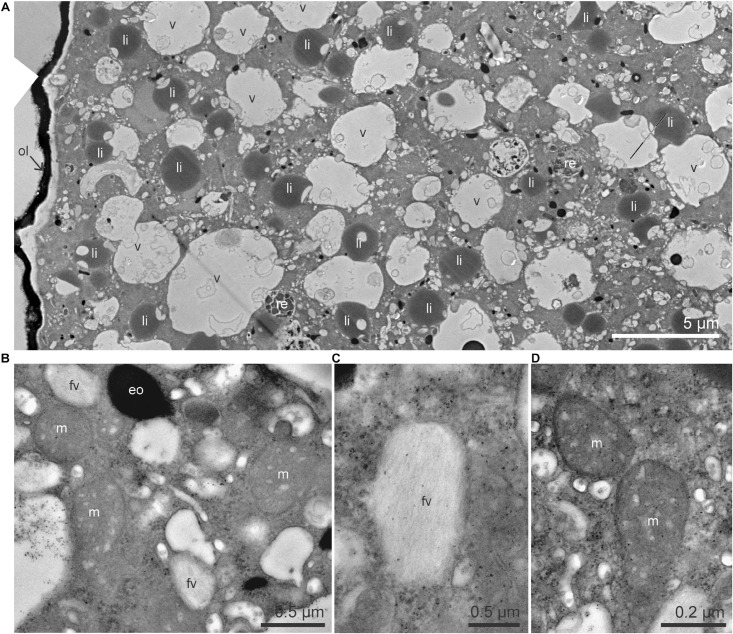
TEM micrographs of the cytoplasm of *Ammonia* sp. **(A)** Global view of the cytoplasm with numerous lipid droplets, vacuoles and a few residual bodies. **(B)** Higher magnification image of mitochondria, electron-opaque bodies, and fibrillar vesicles. **(C)** Detailed structure of a fibrillar vesicle, fibrils are organized in parallel. **(D)** Mitochondria with visible intact cristae and double membrane. eo, electron-opaque bodies; fv, fibrillar vesicles; li, lipid droplets; m, mitochondria; ol, organic lining; re, residual bodies; v, vacuoles.

**FIGURE 2:**
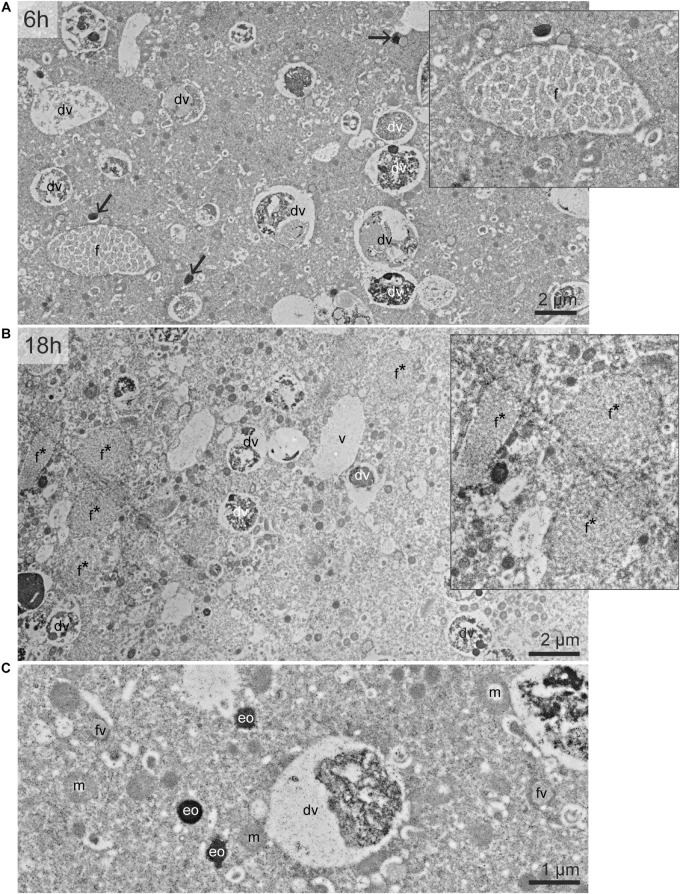
TEM micrographs of the cytoplasm of *G. bulloides.* Views of the cytoplasm in specimen incubated 6 h **(A)** and 18 h **(B)**. **(C)** Detailed structure of fibrillar vesicle, electron-opaque bodies, and mitochondria. Black arrows: electron-opaque bodies (eo). dv, degradation vacuole; f, fibrillar bodies (form B, see text); f*, later stage of fibrillar bodies (form C); fv, fibrillar vesicles; m, mitochondria; v, vacuoles.

The cytoplasm of *Ammonia* sp. exhibited typical organelles observed in other benthic species (LeKieffre et al., 2018a): lipid droplets, organic lining, residual bodies (circular vacuoles with a diameter of about 2–5 μm, containing heterogeneous material), electron-opaque bodies (200–500 nm circular inclusions), fibrillar vesicles (oval vesicles of about 500 nm in length containing fibrils), mitochondria and “empty” vacuoles (from which the content has been lost during sample preparation) (Figure 1). In the *G. bulloides* cytoplasm, degradation vacuoles were relatively abundant and, similarly to *Ammonia* sp., several electron-opaque bodies, fibrillar vesicles (fv), and “empty” vacuoles were also seen (Figure 2). Fibrillar bodies (f) were observed in all observed *G. bulloides*; however, their appearance differed between the individuals incubated for 6 h from those incubated for 18 h (Figure 2). Fibrillar bodies (f) of specimens incubated for 6 h (Figure 2A) had a clearly distinct fibrillar content as in form B described in *G. sacculifer* (Anderson and Bé, 1976; Text Figure 1). On the other hand, the fibrils in the fibrillar bodies (f) observed in specimens incubated for 18 h (Figure 2B) had less distinct fibrillar material, as in form C described by Anderson and Bé (Anderson and Bé, 1976) as “highly dispersed fibrillar material.”

**FIGURE 3:**
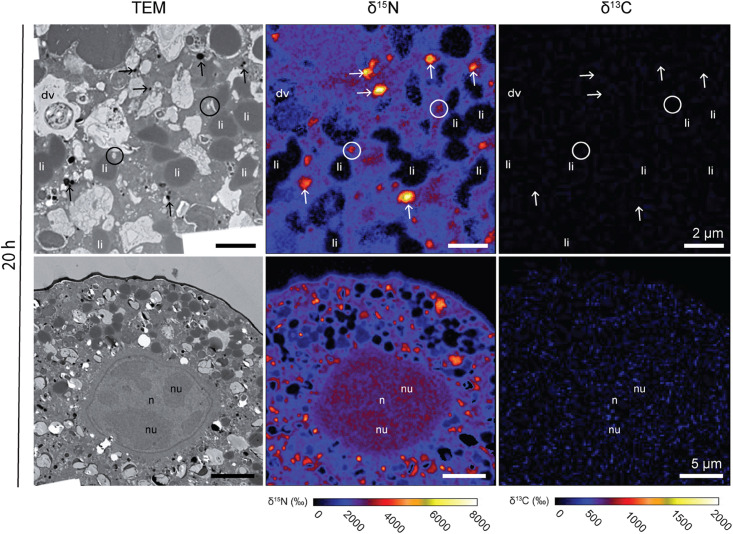
^13^C and ^15^N cellular localization in the cytoplasm of *Ammonia* sp. after 20 h of incubation in light with H^13^CO_3_^–^ and ^15^NH_4_^+^. Left column: TEM micrographs; central column: corresponding NanoSIMS δ^15^N map; right column: corresponding NanoSIMS δ^13^C map. Arrows: electron-opaque bodies; circles: fibrillar vesicles. dv, degradation vacuoles; li, lipid droplets; n, nucleoplasm; nu, nucleus.

**FIGURE 4:**
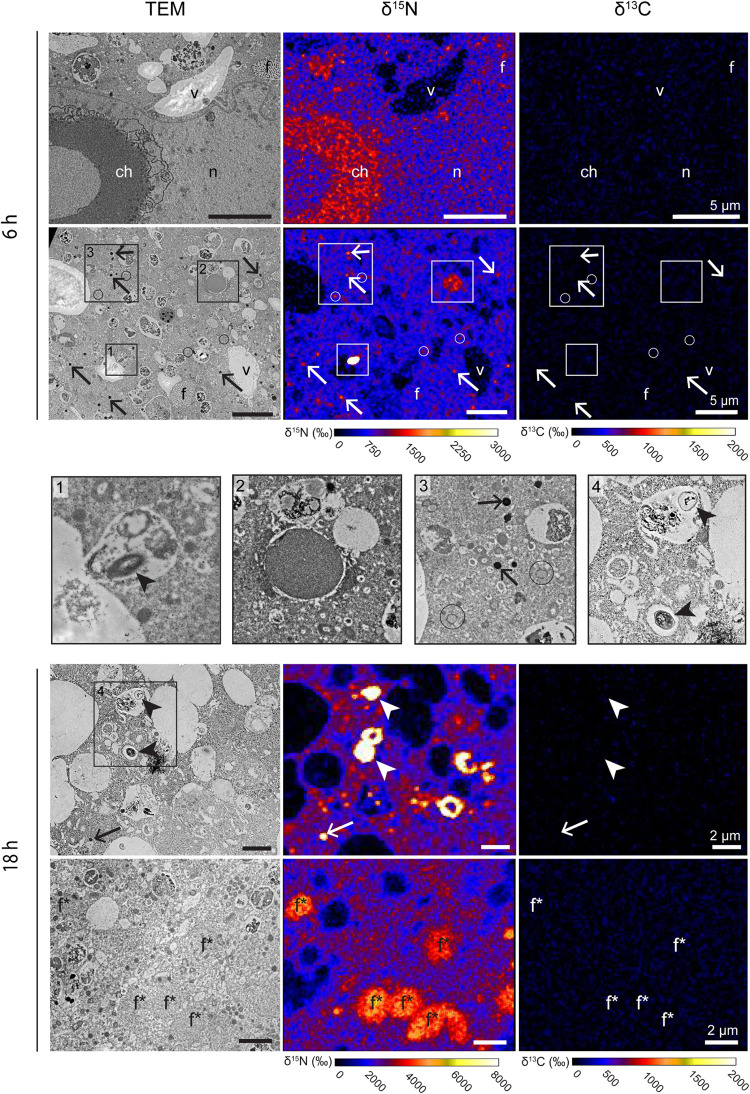
^13^C and ^15^N cellular localization in the cytoplasm of *G. bulloides* after 6 and 18 h of incubation in light with H^13^CO_3_^–^ and ^15^NH_4_^+^. Left column: TEM micrographs; central column: corresponding NanoSIMS δ^15^N map; right column: corresponding NanoSIMS δ^13^C map. Arrows: electron-opaque bodies; arrowheads: prokaryote-like vesicles; circles: fibrillar vesicles. ch, condensed chromatin; dv, degradation vacuoles; f, fibrillar bodies (form B, see text); f*, later stage of fibrillar bodies (form C); li, lipid droplets; n, nucleoplasm; nu, nucleus; v, vacuoles.

In one specimen per species, a nucleus was imaged with TEM and NanoSIMS. The ultrastructure of these nuclei were different from one species to another. In *Ammonia* sp. the nucleus exhibited a typical structure as described in previous studies (LeKieffre et al., 2018a), with a double membrane and several nucleoli scattered in the nucleoplasm (Figure 3). In *G. bulloides*, the nucleus had a double membrane, but no nucleolus in the imaged region, and exhibited a large portion of condensed chromatin (Figure 4; 6 h).

No intact prokaryotic symbionts nor kleptoplasts could be seen in the cytoplasm of *Ammonia* sp. on the TEM micrographs (Figure 1). No intact algal or prokaryotic symbionts were observed in the *G. bulloides* specimens (Figure 2). However, some prokaryote-like vesicles were observed in the cytoplasm of two out of four of the *G. bulloides* incubated during this experiment (see arrowheads in Figure 4). These prokaryote-like vesicles had an oval/ovoid shape, with a length of ca. 500 nm and were always enclosed in degradation vacuoles and found in various states of degradation.

### Inorganic Nitrogen and Carbon Assimilation

^15^N-enrichment was observed in all *Ammonia* sp. and *G. bulloides* specimens observed in this study. In contrast, no ^13^C-enrichment was observed in either species (Figures 3, 4). In *Ammonia* sp., after 20 h of incubation, NanoSIMS imaging revealed ^15^N-accumulation mainly in electron-opaque bodies (see arrows in Figure 3) with ^15^N-enrichment values ranging from ca. 2,500 to 6,000‰ (Figure 3) and fibrillar vesicles (ca. 2,000–4,000‰). The cytoplasm was also labeled with an average ^15^N-enrichment value of ca. 2,500‰, although with strong variability among specimens; two specimens exhibited cytoplasmic ^15^N-enrichment of between 3,200 and 3,400‰, while the third specimen only reached ca. 1,000‰. In one of the *Ammonia* sp. specimens, the nucleus was observed and imaged with the NanoSIMS (Figure 3). Its entire structure was ^15^N-labeled, with the nucleoli being slightly more ^15^N-enriched than the nucleoplasm (ca. 4,800‰ for the nucleoli, vs. ca. 4,600‰ for the nucleoplasm) (Figure 3).

The cytoplasm of all *G. bulloides* specimens incubated with ^15^NH_4_^+^ for 6 and 18 h was ^15^N-enriched, increasing from an average of ca. 1,300‰ after 6 h to ca. 2,400‰ after 18 h (Figure 4). Like the *Ammonia* sp. specimens, the ^15^N-enrichment was variable from specimen to specimen, with cytoplasmic enrichment ranging from ca. 750 to 1,900‰ in individuals incubated for 6 h.

In contrast to *Ammonia* sp., the nucleoplasm of the nucleus imaged in one of the *G. bulloides* specimens (incubated for 6 h with ^15^NH_4_^+^), was ^15^N-enriched to levels similar to the cytoplasm. However, ^15^N-enriched condensed chromatin (ca. 1,200‰, vs. ca. 750‰ for the cytoplasm) was observed (Figure 4). As in *Ammonia* sp., electron-opaque bodies and fibrillar vesicles accumulated ^15^N after 6 h of incubation (Figure 4, panel 3 and Supplementary Figure S2). The enrichment of these features was variable among specimens and over time: from ca. 4000‰ to ca. 11,500‰ for electron-opaque bodies, and ca. 1,000–2,000‰ for fibrillar vesicles (fv). Fibrillar bodies (f) (both forms B and C) were also ^15^N-labeled. A few other unidentified structures were also ^15^N-enriched, e.g., dense vesicles of 2–3 μm of diameter (Figure 4, panel 2). After 6 h, fibrillar body (form B) ^15^N-enrichment was variable, a few fibrillar bodies (f) with cytoplasmic enrichment values could be seen, however, the majority clearly accumulated more ^15^N-labeling than the cytoplasm with values ranging from ca. 1,200 to 4,000‰. After 18 h, all the fibrillar bodies (form C) had increased ^15^N-enrichment values of ca. 4,000–4,600‰, clearly above the cytoplasmic enrichment of ca. 2,400‰. A few unidentified structures observed in *G. bulloides* cytoplasm also accumulated ^15^N-labeling (e.g., Figure 4, panel 2). Finally, in two of the four *G. bulloides* specimens observed in this study, highly ^15^N-enriched hotspots (ca. 16,000–66,000‰) could be seen in the cytoplasm, some corresponding to prokaryote-like vesicles enclosed in degradation vacuoles (Figure 4, panels 1 and 4).

## Discussion

### Heterotrophy and Ammonium Assimilation

Previous observations of chloroplasts in the cytoplasm of *Ammonia* sp. (Jauffrais et al., 2018; Koho et al., 2018) were suggested to result from microalgal food ingestion and slow digestion, rather than active sequestration as their photosynthetic activity quickly decreased when not fed (Jauffrais et al., 2016). We did not observe intact chloroplasts in the specimens studied here, and our NanoSIMS data shows that no ^13^C-labeled carbon is present in the foraminiferal cell, thus confirming a lack of photosynthesis and the heterotrophic nature of *Ammonia* sp.

*G. bulloides* is well-known to be barren of algal symbionts, which was corroborated in the TEM images of our *G. bulloides* samples. Despite the report of Bird et al. (2017) highlighting the presence of *Synechococcus* endobionts, these were not observed in the specimens studied here. A lack of both algal symbionts and cyanobacterial endobionts indicates a heterotrophic mode of feeding in the *G. bulloides* specimens investigated in this study. The lack of observable ^13^C-enrichment in the cytoplasm of *G. bulloides* after 18 h exposure to H^13^CO_3_ (Figure 4) supports this conclusion.

In contrast to the ^13^C incubation results, after incubation with ^15^N-ammonium, much of the *Ammonia* sp. and *G. bulloides* cytosol was enriched in ^15^N. Furthermore, two different structures were particularly enriched, the electron opaque bodies and fibrillar vesicles (Figures 3, 4). Both are common organelles in benthic foraminifera (LeKieffre et al., 2018a). Electron opaque bodies have been previously described in both benthic and planktonic foraminifera as electron-dense bodies or osmiophilic granules (Leutenegger, 1977; Nomaki et al., 2016; LeKieffre et al., 2018a). In *H. germanica*, an intertidal benthic foraminiferal species quite similar in morphology to *Ammonia* sp., the electron opaque bodies became enriched in ^15^N and ^13^C after incubation with ^15^NH_4_Cl and H^13^CO_3_ (LeKieffre et al., 2018b). In this study of *Ammonia* sp., these subcellular compartments became enriched in ^15^N but, as expected not in ^13^C (Figure 3). In addition, Nomaki et al. (2016) also demonstrated that in *Ammonia* sp. the electron opaque bodies are enriched in ^34^S after incubation with Na_2_^34^SO_4_ under dysoxic conditions (0.1–22 μmol l^–1^ O_2_). They suggested that electron opaque bodies play a role in N-assimilation in oxygen-depleted environments due to their ^15^N-enrichment under both dysoxic and anoxic conditions (compared to ^34^S-enrichment only under dysoxic conditions). However, our study shows that the electron opaque bodies are also abundant and ^15^N-enriched under oxic conditions, indicating that their ^15^N-enrichment is not a function of oxygen concentration. Interestingly, the distribution of these electron opaque bodies may be a function of oxygen availability, as this differs between oxic conditions, where there is dispersal across the cell (this study, Koho et al., 2018) and anoxic conditions where they aggregate close to the cell periphery (Nomaki et al., 2016; Koho et al., 2018). However, the purpose of these electron-opaque bodies remains unclear.

Fibrillar vesicles (fv) are thought to be involved in the transport of sulfated amino-polysaccharides called glycosaminoglycans (GAGs) (Langer, 1992). Thus, an enrichment in ^15^N is not surprising. GAGs are transported in vesicles to the cell membrane for formation into the organic lining or are secreted and are a significant component of the organic matrix template for calcium precipitation (Weiner and Erez, 1984; Langer, 1992), thereby playing a fundamental role in the growth of the foraminifera.

Of significance is the ^15^N-enrichment of nuclear material observed in both *Ammonia* sp. and *G. bulloides*. This nuclear material includes N-rich proteins and DNA and their enrichment in ^15^N indicate the assimilation of ammonium-derived N into the cell infrastructure. The fibrillar bodies (f) in planktonic species, which also became enriched in ^15^N, are proteinaceous organelles (Lee et al., 1965). Spero (1988) used TEM imaging to build the hypothesis that the fibrillar body proteins are involved in forming the organic matrix at the site of calcification, and hence are vital to the ontogenetic growth of the foraminifera. In our experiment, *G. bulloides* specimens were exposed to relatively high N concentrations (10 μM ^15^NH_4_Cl) compared to the low ambient concentrations in surface waters, where *Ammonia* is rapidly recycled and consequently concentrations are often below detection levels (Mulholland and Lomas, 2008). In our experiment, the increased availability of ammonium to *G. bulloides* could have led to increased protein production and storage within the fibrillar bodies (f), resulting in their higher enrichment and the shift in appearance from form B at 6 h to form C at 18 h (cf. Figures 2A,B).

Our observations indicate that N-assimilation occurs in similar organelles in both planktonic and benthic species (except fibrillar bodies, absent from benthic foraminifera) that are crucial to foraminiferal growth and development.

### Pathways of Ammonium Assimilation

The small number of prokaryote-like vesicles in the TEM images of two of the four *G. bulloides* specimens (including in additional TEM sections not processed through to NanoSIMS) were observed enclosed in degradation vacuoles and were in various states of degradation (Arrowheads, Figure 4). It is probable that they are bacterial prey (Bird et al., 2017) and could be a source of ^15^N-enrichment if exposed to ^15^N-ammonium prior to phagocytosis. A potential source of bacteria might have been the microenvironment formed around the spines of *G. bulloides* by its rhizopodial network, as bacteria could remain within this throughout the picking process. However, during the 2-day recovery phase in 0.2 μm filter-sterilized seawater (FSW), many of the microenvironment bacteria would have been phagocytosed and digested by the foraminifera prior to transfer to the experimental vial containing spiked 0.2 μm FSW. Nevertheless, it is reasonable to assume that some phagocytosis of ^15^N-enriched bacteria could occur. To understand the potential contribution of phagocytosed bacteria to ^15^N-enrichment of the foraminiferal cell, the same experimental procedures, but using ^15^N-NO_3_ as the N-source, could be carried out. Planktic foraminifera are incapable of nitrate assimilation into their biomass (LeKieffre et al., 2020) and therefore any resulting ^15^N-enrichment in the foraminiferal cell must come from contaminating bacteria. LeKieffre et al. (2020) exposed *O. universa* to ^15^N-NO_3_ following the same experimental procedures used here. Their results showed prokaryote-like vesicles in degradation vacuoles highly enriched in ^15^NO_3_, whilst the cytoplasm and organelles of the foraminiferal cell, by contrast, exhibited extremely low ^15^N-labeling, within the range of the control values after 6 h of incubation, and barely above the control values after 18 h. This indicates that the limited numbers of highly enriched bacterial prey available to the foraminifera in these experiments are not the major source of cytosol and organelle ^15^N-enrichment. If we assume that a single exponentially growing marine bacterium contains 35 fg N (Vrede et al., 2002) and an average foraminiferal specimen contains 219 ng N (Enge et al., 2018) it would require more than 10^9^ bacteria to supply all the N needs of the foraminiferal cell. Therefore, we conclude that the ^15^N-enrichment observed in the cytoplasm of *G. bulloides* and *Ammonia* sp. was predominantly due to direct ^15^N-ammonium assimilation by the foraminifera, rather than through digestion of ^15^N-labeled bacteria, although a small contribution from the latter cannot be excluded.

*Ammonia* sp. and *G. bulloides* appear to assimilate ammonium for growth and development without the use of the chloroplast-based GS/GOGAT pathway available in photosynthesizing symbionts or kleptoplasts (Alipanah et al., 2015; Sanz-Luque et al., 2015). Therefore, these heterotrophic protists must have an innate cellular pathway for inorganic ammonium assimilation, which could be driven by a mitochondrial-based GDH pathway and/or a cytosol-based GS/GOGAT pathway. In the absence of any complete or partial searchable genomes in the marine foraminifera, we performed a protein search for GDH, GS, and GOGAT within the only annotated foraminiferal genome currently available at NCBI; that of the fresh water species *Reticulomyxa filosa* (Assembly GCA_000512085.1; Glöckner et al., 2014). The GDH protein search yielded an NAD-dependent glutamate dehydrogenase protein (NAD-GDH; GenBank accession ETO29704.1). GDH is found in all organisms, it links carbon and nitrogen metabolism, by catalyzing both the catabolic oxidative deamination of glutamate to α-ketoglutarate and ammonia, and the reverse anabolic reductive amination of α-ketoglutarate to glutamate (i.e., ammonium assimilation) (Hudson and Daniel, 1993). Although there is some debate about the predominant directionality of the reaction, the high K_*m*_ of GDH for ammonium may prohibit ammonium assimilation, under normal cellular conditions in most eukaryotic organisms (Li et al., 2011; van Heeswijk et al., 2013). GDH is found in three basic types specific for different co-factors: NAD-dependent, NADP-dependent and NAD/NADP-dependent (Hudson and Daniel, 1993). In micro-organisms, some evidence suggests that the catabolic reaction producing α-ketoglutarate is largely carried out by NAD-GDH, and the anabolic ammonium assimilation reaction is predominantly carried out by NADP-GDH, although there are exceptions (Miller and Magasanik, 1990; Chavez and Candau, 1991; Hudson and Daniel, 1993). The identification of an NAD-GDH in *R. filosa*, hints at a role in the catabolic oxidative deamination of glutamate rather than in ammonium assimilation. However, characterization of this enzyme, and an understanding of gene regulation would be necessary to fully determine directionality in this organism. The GS protein search yielded a GS catalytic region protein (GenBank accession ETO36073.1) with around 50% identity with GS from several bacteria from the phylum bacteriodetes of the genus *Dyadobacter* (e.g., *Dyadobacter tibetensis*, 52.7% identity, accession number WP_025763368). The GOGAT protein search yielded a partial (200 amino acids) putative NAD(P)H-dependent glutamate synthase (GenBank accession ETO26634.1). However, a BLASTp search with this putative GOGAT enzyme sequence produced no matches indicating a lack of similarity with other GOGAT enzymes. Nevertheless, the presence of a GS catalytic region and a putative GOGAT indicates that, in addition to GDH, the freshwater *R. filosa* potentially houses the alternative, more energetically expensive, but higher affinity GS/GOGAT ammonium assimilation pathway. Finally, we performed a protein search for an ammonium transporter in the *R. filosa* genome revealing both a putative Amt transporter (Genbank accession ET023911.1) and a putative Rhesus ammonium transporter (Genbank accession ETO07715.1). The Amt transporter (found in bacteria, archaea, fungi, and plants) and its analogs, methylammonium permease (yeast) and Rhesus proteins (animals) are a family of integral membrane proteins, found in nearly all organisms. They carry out the high affinity and highly selective movement of ammonium across biological membranes (Pantoja, 2012). The presence of the putative AmtB, Rhesus type-A, GDH and GS in *R. filosa* alongside our NanoSIMS evidence, suggests that assimilation of ammonium may be found across freshwater, benthic and planktonic foraminifera.

The role of inorganic N-assimilation in these heterotrophic protists may be as a supplementary to food ingestion when inorganic ammonium availability is high. Ammonium availability will be sporadic for the planktonic *G. bulloides* in the open ocean habitat, and perhaps one function of the N-enriched fibrillar bodies is as a proteinaceous N store under N-replete conditions. Whether *Ammonia* sp. and *G. bulloides* harbor and utilize one or both assimilatory pathways is yet to be determined, and indeed, they may use different pathways given the difference in the concentration of ammonium in the open ocean vs. coastal sediments (e.g., 38). Once more genetic information is available for foraminfera, an investigation of the ammonium-assimilation genes in marine foraminifera is required to determine the pathway, the prevalence and the genetic and environmental control of this system in these heterotrophic protists.

### The Marine Nitrogen Cycle

The microbial loop, first proposed by Azam et al. (1983), is a model of the system of carbon and nutrient cycling through the microbial components of the marine ecosystem. It channels particulate and dissolved carbon and nutrients (e.g., organic and inorganic N) via bacteria to heterotrophic protists, and into the wider food web via larger zooplankton, fish and cetaceans. The ability of heterotrophic protists to utilize DIN reveals an additional pathway within the microbial loop for N-assimilation into the food web. Heterotrophic protists are ubiquitous (Pernice et al., 2015; Mangot et al., 2018) and their functionality diverse (Sherr et al., 2007; Caron et al., 2012; Mitra et al., 2016). Many phototrophic species contribute to primary production and inorganic nutrient uptake, whilst others like *Ammonia* sp. and *G. bulloides* act at a variety of trophic levels through their diets of bacteria, phytoplankton and metazoans (Bird et al., 2017; Chronopoulou et al., 2019). Despite the bioavailability of ammonium (compared to N_2_ for example) and the ubiquity of heterotrophic protists, the across-taxa ability to assimilate ammonium is currently unknown. In addition, cell-specific ammonium uptake rates are rarely documented (Klawonn et al., 2019). Yet, taxa-specific nutrient preferences, assimilation rates and quantitatively important taxa for ammonium cycling are all important factors in understanding ecosystem functioning and biogeochemical cycling. These in turn, are dependent on the characteristics of the individual species present, the assemblage diversity and shifts in time and space driven by environmental parameters (Piredda et al., 2017).

## Conclusion

In conclusion, using TEM and NanoSIMS we demonstrate that two heterotrophic protists from contrasting marine habitats have an innate pathway for ammonium assimilation into organelles crucial to growth and development, indicating that heterotrophy is not the only source of N for these organisms. This provides evidence that the observed ammonium assimilation in the mixotrophic foraminiferal species, *Orbulina universa* (LeKieffre et al., 2020) and two benthic kleptoplastic species (LeKieffre et al., 2018b; Jauffrais et al., 2019), could be carried out by the foraminifera themselves rather than the symbionts. In addition, a GenBank search has highlighted putative ammonium assimilation proteins; the ammonium transporters AmtB and Rhesus Type-A and glutamate dehydrogenase, annotated in the genome of the freshwater foraminifer *R. filosa*. This suggests that inorganic ammonium assimilation may potentially occur in both freshwater and marine foraminifera. Additional work to study the genes involved in ammonium assimilation in the foraminifera, and the environmental conditions under which uptake of ammonium occurs need to be carried out. In addition, the ammonium assimilation pathway should be investigated in other heterotrophic protists to begin to understand the contribution of this newly revealed pathway to the microbial loop and ecosystem functioning.

## Data Availability Statement

All datasets generated for this study are included in the article/Supplementary Material, further inquiries can be directed to the corresponding author.

## Author Contributions

CB and CL devised and carried out the experiments and wrote the manuscript. TJ, EG, HF, AM, and OM provided advice, resources, and material for working on benthic foraminifera. EG and AM provided guidance on experimental set up of benthic experiments. AR and JF provided resources and expertise and assisted in collection and culturing of the planktonic foraminifera. All authors contributed to the writing and editing of the manuscript.

## Conflict of Interest

The authors declare that the research was conducted in the absence of any commercial or financial relationships that could be construed as a potential conflict of interest.

## Supplementary Material

Supplementary Figure 1 Representative ion counts for the ions ^12^C^12^C^–^, ^13^C^12^C^–^-, ^12^C^14^N^–^, and ^12^C^15^N^–^ for the species *Ammonia* sp. and *G. bulloides* obtained after drift correction and accumulation of the six to eight sequential images acquired using the software L’IMAGE.

Supplementary Figure 2 ^15^N cellular localization in the electron-opaque bodies and fibrillar vesicles of *G. bulloides* after 6 h. Arrows: electron-opaque bodies; circles: fibrillar vesicles.

Supplementary Table 1 Sampling information and experimental manipulations for each foraminiferal specimen collected.

Supplementary Table 2 Excel spreadsheet of raw data with means and SDs of natural isotopic δ^13^C and δ^15^N values and ^15^N enrichments in regions of interest (ROIs) in different sub-cellular structures within *G. bulloides* and *Ammonia* sp. ROIs were drawn on the NanoSIMS images with the software L’IMAGE to quantify mean ^15^N enrichments of a given foraminifera. Note that control values were measured on *Ammonia* sp. and *Orbulina universa* species.

## References

[B1] Alipanah L. Rohloff J. Winge P. Bones A. M. Brembu T. (2015). Whole-cell response to nitrogen deprivation in the diatom *Phaeodactylum tricornutum*. *J. Exp. Bot.* 66 6281–6296. 26163699 10.1093/jxb/erv340 PMC4588885

[B2] Anderson O. R. Bé A. W. H. (1976). The ultrastructure of a planktonic foraminifer, *Globigerinoides sacculifer* (Brady), and its symbiotic dinoflagellates. *J. Foraminifer. Res.* 6 1–21. 10.2113/gsjfr.6.1.1 28159795

[B3] Azam F. Fenchel T. Field J. G. Grey J. S. Meyer-Reil L. A. Thingstad F. (1983). The ecological role of water-column microbes. *Mar. Ecol. Prog. Ser.* 10 257–263. 10.3354/meps010257

[B4] Bird C. Darling K. F. Russell A. D. Davis C. V. Fehrenbacher J. Free A. (2017). Cyanobacterial endobionts within a major marine planktonic calcifier (*Globigerina bulloides*, Foraminifera) revealed by 16S rRNA metabarcoding. *Biogeosciences* 14 901–920. 10.5194/bg-14-901-2017

[B5] Bird C. Darling K. F. Russell A. D. Fehrenbacher J. S. Davis C. V. Free A. (2018). 16S rRNA gene metabarcoding and TEM reveals different ecological strategies within the genus Neogloboquadrina (planktonic foraminifer). *PLoS One* 13:e0191653. 10.1371/journal.pone.0191653 29377905 PMC5788372

[B6] Bird C. Schweizer M. Roberts A. Austin W. E. Knudsen K. L. Evans K. M. (2020). The genetic diversity, morphology, biogeography, and taxonomic designations of *Ammonia* (Foraminifera) in the Northeast Atlantic. *Mar. Micropaleontol.* 155:101726 10.1016/j.marmicro.2019.02.001

[B7] Bond N. A. Cronin M. F. Freeland H. Mantua N. (2015). Causes and impacts of the 2014 warm anomaly in the NE Pacific. *Geophys. Res. Lett.* 42 3414–3420. 10.1002/2015gl063306

[B8] Caron D. A. Countway P. D. Jones A. C. Kim D. Y. Schnetzer A. (2012). Marine protistan diversity. *Annu. Rev. Mar. Sci.* 4 467–493. 10.1146/annurev-marine-120709-142802 22457984

[B9] Chavez S. Candau P. (1991). An NAD-specific glutamate dehydrogenase from cyanobacteria identification and properties. *FEBS Lett.* 285 35–38. 10.1016/0014-5793(91)80719-j 1906012

[B10] Chronopoulou P.-M. Salonen I. Bird C. Reichart G.-J. Koho K. A. (2019). Metabarcoding insights into the trophic behavior and identity of intertidal benthic foraminifera. *Front. Microbiol.* 10:1169. 10.3389/fmicb.2019.01169 31191490 PMC6547873

[B11] Darling K. F. Wade C. M. (2008). The genetic diversity of planktic foraminifera and the global distribution of ribosomal RNA genotypes. *Mar. Micropaleontol.* 67 216–238. 10.1016/j.marmicro.2008.01.009

[B12] Dupuy C. Rossignol L. Geslin E. Pascal P.-Y. (2010). Predation of mudflat meio-macrofaunal metazoans by a calcareous foraminifer, *Ammonia tepida* (Cushman, 1926). *J. Foraminifer. Res.* 40 305–312. 10.2113/gsjfr.40.4.305 28159795

[B13] Enge A. J. Wanek W. Heinz P. (2018). Preservation effects on isotopic signatures in benthic foraminiferal biomass. *Mar. Micropaleontol.* 144 50–59. 10.1016/j.marmicro.2018.09.002

[B14] Enge A. J. Wukovits J. Wanek W. Watzka M. Witte U. F. M. Hunter W. R. (2016). Carbon and nitrogen uptake of calcareous benthic foraminifera along a depth-related oxygen gradient in the OMZ of the Arabian Sea. *Front. Microbiol.* 7:71. 10.3389/fmicb.2016.00071 26903959 PMC4749719

[B15] Field D. B. (2004). Variability in vertical distributions of planktonic foraminifera in the California current: relationships to vertical ocean structure. *Paleoceanography* 19:PA2014.

[B16] Gastrich M. D. (1987). Ultrastructure of a new intracellular symbiotic alga found within planktonic foraminifera. *J. Phycol.* 23 623–632. 10.1111/j.1529-8817.1987.tb04215.x

[B17] Gibbin E. Banc-Prandi G. Fine M. Comment A. Meibom A. (2020). A method to disentangle and quantify host anabolic turnover in photosymbiotic holobionts with subcellular resolution. *Commun. Biol.* 3:14. 10.1038/s42003-019-0742-6 31925332 PMC6949218

[B18] Glock N. Roy A.-S. Romero D. Wein T. Weissenbach J. Revsbech N. P. (2019). Metabolic preference of nitrate over oxygen as an electron acceptor in foraminifera from the Peruvian oxygen minimum zone. *Proc. Natl. Acad. Sci. U.S.A.* 116 2860–2865. 10.1073/pnas.1813887116 30728294 PMC6386669

[B19] Glock N. Schönfeld J. Eisenhauer A. Hensen C. Mallon J. Sommer S. (2013). The role of benthic foraminifera in the benthic nitrogen cycle of the Peruvian oxygen minimum zone. *Biogeosciences* 10 4767–4783. 10.5194/bg-10-4767-2013

[B20] Glöckner G. Hülsmann N. Schleicher M. Noegel A. A. Eichinger L. Gallinger C. (2014). The genome of the foraminiferan *Reticulomyxa filosa*. *Curr. Biol.* 24 11–18. 10.1016/j.cub.2013.11.027 24332546

[B21] Gruber N. (2008). The marine nitrogen cycle: overview and challenges. *Nitrogen Mar. Environ.* 2 1–50. 10.1016/b978-0-12-372522-6.00001-3

[B22] Høgslund S. Revsbech N. P. Cedhagen T. Nielsen L. P. Gallardo V. A. (2008). Denitrification, nitrate turnover, and aerobic respiration by benthic foraminiferans in the oxygen minimum zone off Chile. *J. Exp. Mar. Biol. Ecol.* 359 85–91. 10.1016/j.jembe.2008.02.015

[B23] Holzmann M. Pawlowski J. (2000). Taxonomic relationships in the genus Ammonia (Foraminifera) based on ribosomal DNA sequences. *J. Micropalaeontol.* 19 85–95. 10.1144/jm.19.1.85

[B24] Hönisch B. Bijma J. Russell A. D. Spero H. J. Palmer M. R. Zeebe R. E. (2003). The influence of symbiont photosynthesis on the boron isotopic composition of foraminifera shells. *Mar. Micropaleontol.* 49 87–96. 10.1016/s0377-8398(03)00030-6

[B25] Hudson R. C. Daniel R. M. (1993). l-glutamate dehydrogenases: distribution, properties and mechanism. *Comp. Biochem. Physiol. B Comp. Biochem.* 106 767–792. 10.1016/0305-0491(93)90031-Y 8299344

[B26] Jauffrais T. Jesus B. Metzger E. Mouget J.-L. Jorissen F. Geslin E. (2016). Effect of light on photosynthetic efficiency of sequestered chloroplasts in intertidal benthic foraminifera (*Haynesina germanica* and *Ammonia tepida*). *Biogeosciences* 13 2715–2726. 10.5194/bg-13-2715-2016

[B27] Jauffrais T. LeKieffre C. Koho K. A. Tsuchiya M. Schweizer M. Bernhard J. M. (2018). Ultrastructure and distribution of kleptoplasts in benthic foraminifera from shallow-water (photic) habitats. *Mar. Micropaleontol.* 138 46–62. 10.1016/j.marmicro.2017.10.003

[B28] Jauffrais T. LeKieffre C. Schweizer M. Geslin E. Metzger E. Bernhard J. M. (2019). Kleptoplastidic benthic foraminifera from aphotic habitats: insights into assimilation of inorganic C, N and S studied with sub-cellular resolution. *Environ. Microbiol.* 21 125–141. 10.1111/1462-2920.14433 30277305

[B29] Katz M. E. Cramer B. S. Franzese A. Hönisch B. Miller K. G. Rosenthal Y. (2010). Traditional and emerging geochemical proxies in foraminifera. *J. Foraminifer. Res.* 40 165–192. 10.2113/gsjfr.40.2.165 28159795

[B30] Klawonn I. Bonaglia S. Whitehouse M. J. Littmann S. Tienken D. Kuypers M. M. M. (2019). Untangling hidden nutrient dynamics: rapid ammonium cycling and single-cell ammonium assimilation in marine plankton communities. *ISME J.* 13 1960–1974. 10.1038/s41396-019-0386-z 30911131 PMC6776039

[B31] Kleijne A. Kroon D. Zevenboom W. (1989). Phytoplankton and foraminiferal frequencies in northern Indian Ocean and Red Sea surface waters. *Neth. J. Sea Res.* 24 531–539. 10.1016/0077-7579(89)90131-2

[B32] Koho K. A. LeKieffre C. Nomaki H. Salonen I. Geslin E. Mabilleau G. (2018). Changes in ultrastructural features of the foraminifera *Ammonia* spp. in response to anoxic conditions: field and laboratory observations. *Mar. Micropaleontol.* 138 72–82. 10.1016/j.marmicro.2017.10.011

[B33] Kuypers M. M. Marchant H. K. Kartal B. (2018). The microbial nitrogen-cycling network. *Nat. Rev. Microbiol.* 16 263–276. 10.1038/nrmicro.2018.9 29398704

[B34] Langer M. R. (1992). Biosynthesis of glycosaminoglycans in foraminifera: a review. *Mar. Micropaleontol.* 19 245–255. 10.1016/0377-8398(92)90031-E

[B35] Lee J. J. Freudenthal H. D. Kossoy V. Bé A. W. H. (1965). Cytological observations on two planktonic foraminifera, *Globigerina bulloides* d’Orbigny, 1826, and *Globigerinoides ruber* (d’Orbigny, 1839) Cushman, 1927. *J. Protozool.* 12 531–542. 10.1111/j.1550-7408.1965.tb03253.x

[B36] LeKieffre C. Bernhard J. M. Mabilleau G. Filipsson H. L. Meibom A. Geslin E. (2018a). An overview of cellular ultrastructure in benthic foraminifera: new observations of rotalid species in the context of existing literature. *Mar. Micropaleontol.* 138 12–32. 10.1016/j.marmicro.2017.10.005

[B37] LeKieffre C. Jauffrais T. Geslin E. Jesus B. Bernhard J. M. Giovani M.-E. (2018b). Inorganic carbon and nitrogen assimilation in cellular compartments of a benthic kleptoplastic foraminifer. *Sci. Rep.* 8:10140. 10.1038/s41598-018-28455-1 PMC6031614 29973634

[B38] LeKieffre C. Spero H. J. Russell A. D. Fehrenbacher J. S. Geslin E. Meibom A. (2018c). Assimilation, translocation, and utilization of carbon between photosynthetic symbiotic dinoflagellates and their planktic foraminifera host. *Mar. Biol.* 165:104 10.1007/s00227-018-3362-7

[B39] LeKieffre C. Spangenberg J. E. Mabilleau G. Escrig S. Meibom A. Geslin E. (2017). Surviving anoxia in marine sediments: the metabolic response of ubiquitous benthic foraminifera (*Ammonia tepida*). *PLoS One* 12:e0177604. 10.1371/journal.pone.0177604 28562648 PMC5451005

[B40] LeKieffre C. Spero H. J. Fehrenbacher J. S. Russell A. D. Ren H. Geslin E. (2020). Ammonium is the preferred source of nitrogen for planktonic foraminifer and their dinoflagellate symbionts. *Proc. R. Soc. B Biol. Sci.* 287:20200620. 10.1098/rspb.2020.0620 32546098 PMC7329048

[B41] Leutenegger S. (1977). Ultrastructure de foraminifères perforés et imperforés ainsi que de leurs symbiotes. *Cah. Micropaléontol.* 3 1–52.

[B42] Li M. Li C. Allen A. Stanley C. A. Smith T. J. (2011). The structure and allosteric regulation of glutamate dehydrogenase. *Neurochem. Int.* 59 445–455. 10.1016/j.neuint.2010.10.017 21070828 PMC3135769

[B43] Loussert-Fonta C. Toullec G. Paraecattil A. A. Jeangros Q. Krueger T. Escrig S. (2020). Correlation of fluorescence microscopy, electron microscopy, and NanoSIMS stable isotope imaging on a single tissue section. *Commun. Biol.* 3:362. 10.1038/s42003-020-1095-x PMC7347930 32647198

[B44] Mangot J.-F. Forn I. Obiol A. Massana R. (2018). Constant abundances of ubiquitous uncultured protists in the open sea assessed by automated microscopy. *Environ. Microbiol.* 20 3876–3889. 10.1111/1462-2920.14408 30209866

[B45] Miller S. M. Magasanik B. (1990). Role of NAD-linked glutamate dehydrogenase in nitrogen metabolism in *Saccharomyces cerevisiae*. *J. Bacteriol.* 172 4927–4935. 10.1128/jb.172.9.4927-4935.1990 1975578 PMC213147

[B46] Mitra A. Flynn K. J. Tillmann U. Raven J. A. Caron D. Stoecker D. K. (2016). Defining planktonic protist functional groups on mechanisms for energy and nutrient acquisition: incorporation of diverse mixotrophic strategies. *Protist* 167 106–120. 10.1016/j.protis.2016.01.003 26927496

[B47] Moodley L. Boschker H. T. Middelburg J. J. Pel R. Herman P. M. De Deckere E. (2000). Ecological significance of benthic foraminifera: 13C labelling experiments. *Mar. Ecol. Prog. Ser.* 202 289–295. 10.3354/meps202289

[B48] Mulholland M. R. Lomas M. W. (2008). “Nitrogen uptake and assimilation,” in *Nitrogen in the Marine Environment*, 2nd Edn, eds Capone D. Bronk D. Mulholland M. Carpenter E. (New York, NY: Academic Press), 303–384. 10.1016/b978-0-12-372522-6.00007-4

[B49] Murray J. W. (2006). *Ecology and Applications of Benthic Foraminifera.* Cambridge: Cambridge University Press.

[B50] Naidu P. D. Malmgren B. A. (1996). Relationship between late quaternary upwelling history and coiling properties of *Neogloboquadrina pachyderma* and *Globigerina bulloides* in the Arabian Sea. *J. Foraminifer. Res.* 26 64–70. 10.2113/gsjfr.26.1.64 28159795

[B51] Nomaki H. Bernhard J. M. Ishida A. Tsuchiya M. Uematsu K. Tame A. (2016). Intracellular isotope localization in *Ammonia* sp. (Foraminifera) of oxygen-depleted environments: results of nitrate and sulfate labeling experiments. *Front. Microbiol.* 7:163. 10.3389/fmicb.2016.00163 26925038 PMC4759270

[B52] Nomaki H. LeKieffre C. Escrig S. Meibom A. Yagyu S. Richardson E. A. (2018). Innovative TEM-coupled approaches to study foraminiferal cells. *Mar. Micropaleontol.* 138 90–104. 10.1016/j.marmicro.2017.10.002

[B53] Pantoja O. (2012). High affinity ammonium transporters: molecular mechanism of action. *Front. Plant Sci.* 3:34. 10.3389/fpls.2012.00034 22645581 PMC3355798

[B54] Pascal P.-Y. Dupuy C. Richard P. Niquil N. (2008). Bacterivory in the common foraminifer *Ammonia tepida*: isotope tracer experiment and the controlling factors. *J. Exp. Mar. Biol. Ecol.* 359 55–61. 10.1016/j.jembe.2008.02.018

[B55] Pernice M. C. Forn I. Gomes A. Lara E. Alonso-Sáez L. Arrieta J. M. (2015). Global abundance of planktonic heterotrophic protists in the deep ocean. *ISME J.* 9 782–792. 10.1038/ismej.2014.168 25290506 PMC4331586

[B56] Piña-Ochoa E. Hogslund S. Geslin E. Cedhagen T. Revsbech N. P. Nielsen L. P. (2010). Widespread occurrence of nitrate storage and denitrification among Foraminifera and *Gromiida*. *Proc. Natl. Acad. Sci. U.S.A.* 107 1148–1153. 10.1073/pnas.0908440107 20080540 PMC2824274

[B57] Piredda R. Tomasino M. P. D’Erchia A. M. Manzari C. Pesole G. Montresor M. (2017). Diversity and temporal patterns of planktonic protist assemblages at a Mediterranean long term ecological research site. *FEMS Microbiol. Ecol.* 93:fiw200. 10.1093/femsec/fiw200 27677681

[B58] Risgaard-Petersen N. Langezaal A. M. Ingvardsen S. Schmid M. C. Jetten M. S. M. Op den Camp H. J. M. (2006). Evidence for complete denitrification in a benthic foraminifer. *Nature* 443 93–96. 10.1038/nature05070 16957731

[B59] Sanz-Luque E. Chamizo-Ampudia A. Llamas A. Galvan A. Fernandez E. (2015). Understanding nitrate assimilation and its regulation in microalgae. *Front. Plant Sci.* 6:899. 10.3389/fpls.2015.00899 26579149 PMC4620153

[B60] Sautter L. R. Thunell R. C. (1991). Planktonic foraminiferal response to upwelling and seasonal hydrographic conditions; sediment trap results from San Pedro Basin, Southern California Bight. *J. Foraminifer. Res.* 21 347–363. 10.2113/gsjfr.21.4.347 28159795

[B61] Schiebel R. (2002). Planktic foraminiferal sedimentation and the marine calcite budget. *Glob. Biogeochem. Cycles* 16:1065 10.1029/2001GB001459

[B62] Schiebel R. Barker S. Lendt R. Thomas H. Bollmann J. (2007). Planktic foraminiferal dissolution in the twilight zone. *Deep Sea Res. II Top. Stud. Oceanogr.* 54 676–686. 10.1016/j.dsr2.2007.01.009

[B63] Schiebel R. Hemleben C. (eds) (2017). *Planktic Foraminifers in the Modern Ocean.* Berlin: Springer.

[B64] Sherr B. F. Sherr E. B. Caron D. A. Vaulot D. Worden A. Z. (2007). Oceanic protists. *Oceanography* 20 130–134. 10.5670/oceanog.2007.57

[B65] Smart S. M. Ren H. Fawcett S. E. Schiebel R. Conte M. Rafter P. A. (2018). Ground-truthing the planktic foraminifer-bound nitrogen isotope paleo-proxy in the Sargasso Sea. *Geochim. Cosmochim. Acta* 235 463–482. 10.1016/j.gca.2018.05.023

[B66] Spero H. J. (1987). Symbiosis in the Planktonic Foraminifer, *Orbulina universa*, and the isolation of its symbiotic dinoflagellate, *Gymnodinium béii* Sp. Nov.1. *J. Phycol.* 23 307–317. 10.1111/j.1529-8817.1987.tb04139.x

[B67] Spero H. J. (1988). Ultrastructural examination of chamber morphogenesis and biomineralization in the planktonic foraminifer *Orbulina universa*. *Mar. Biol.* 99 9–20. 10.1007/BF00644972

[B68] Spero H. J. Lea D. W. (1996). Experimental determination of stable isotope variability in *Globigerina bulloides*: implications for paleoceanographic reconstructions. *Mar. Micropaleontol.* 28 231–246. 10.1016/0377-8398(96)00003-5

[B69] Thunell R. Sautter L. R. (1992). Planktonic foraminiferal faunal and stable isotopic indices of upwelling: a sediment trap study in the San Pedro Basin, Southern California Bight. *Geol. Soc. Lond. Spec. Publ.* 64 77–91. 10.1144/gsl.sp.1992.064.01.05

[B70] Uhle M. E. Macko S. A. Spero H. J. Engel M. H. Lea D. W. (1997). Sources of carbon and nitrogen in modern planktonic foraminifera: the role of algal symbionts as determined by bulk and compound specific stable isotopic analyses. *Org. Geochem.* 27 103–113. 10.1016/S0146-6380(97)00075-2

[B71] van Heeswijk W. C. Westerhoff H. V. Boogerd F. C. (2013). Nitrogen assimilation in *Escherichia coli*: putting molecular data into a systems perspective. *Microbiol. Mol. Biol. Rev.* 77 628–695. 10.1128/mmbr.00025-13 24296575 PMC3973380

[B72] Vrede K. Heldal M. Norland S. Bratbak G. (2002). Elemental composition (C, N, P) and cell volume of exponentially growing and nutrient-limited bacterioplankton. *Appl. Environ. Microbiol.* 68 2965–2971. 10.1128/aem.68.6.2965-2971.2002 12039756 PMC123973

[B73] Weiner S. Erez J. (1984). Organic matrix of the shell of the foraminifer, *Heterostegina depressa*. *J. Foraminifer. Res.* 14 206–212. 10.2113/gsjfr.14.3.206 28159795

[B74] Woehle C. Roy A.-S. Glock N. Wein T. Weissenbach J. Rosenstiel P. (2018). A novel eukaryotic denitrification pathway in foraminifera. *Curr. Biol.* 28 2536–2543.e5. 10.1016/j.cub.2018.06.027 30078568 PMC6783311

