# Supplementary figures and images for: Heterotrophic Foraminifera Capable of Inorganic Nitrogen Assimilation
Source: Front Microbiol. 2020 Dec 3;11:604979. doi: 10.3389/fmicb.2020.604979 (PMC7744380; doi:10.3389/fmicb.2020.604979)

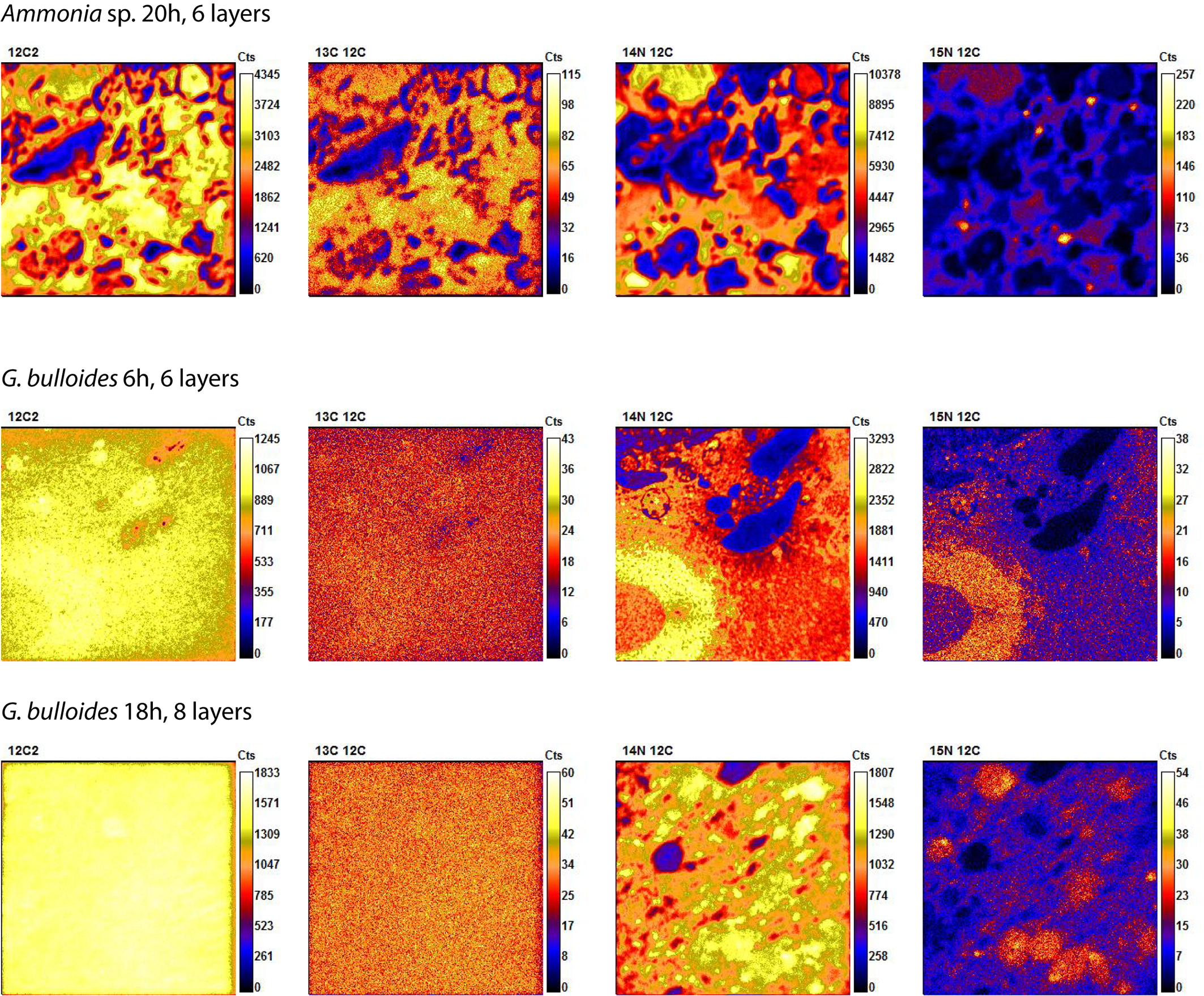

Supplement: Supplementary Figure 1 — Representative ion counts for the ions 12C12C–, 13C12C–-, 12C14N–, and 12C15N– for the species Ammonia sp. and G. bulloides obtained after drift correction and accumulation of the six to eight sequential images acquired using the software L’IMAGE. [file Image_1.TIF]

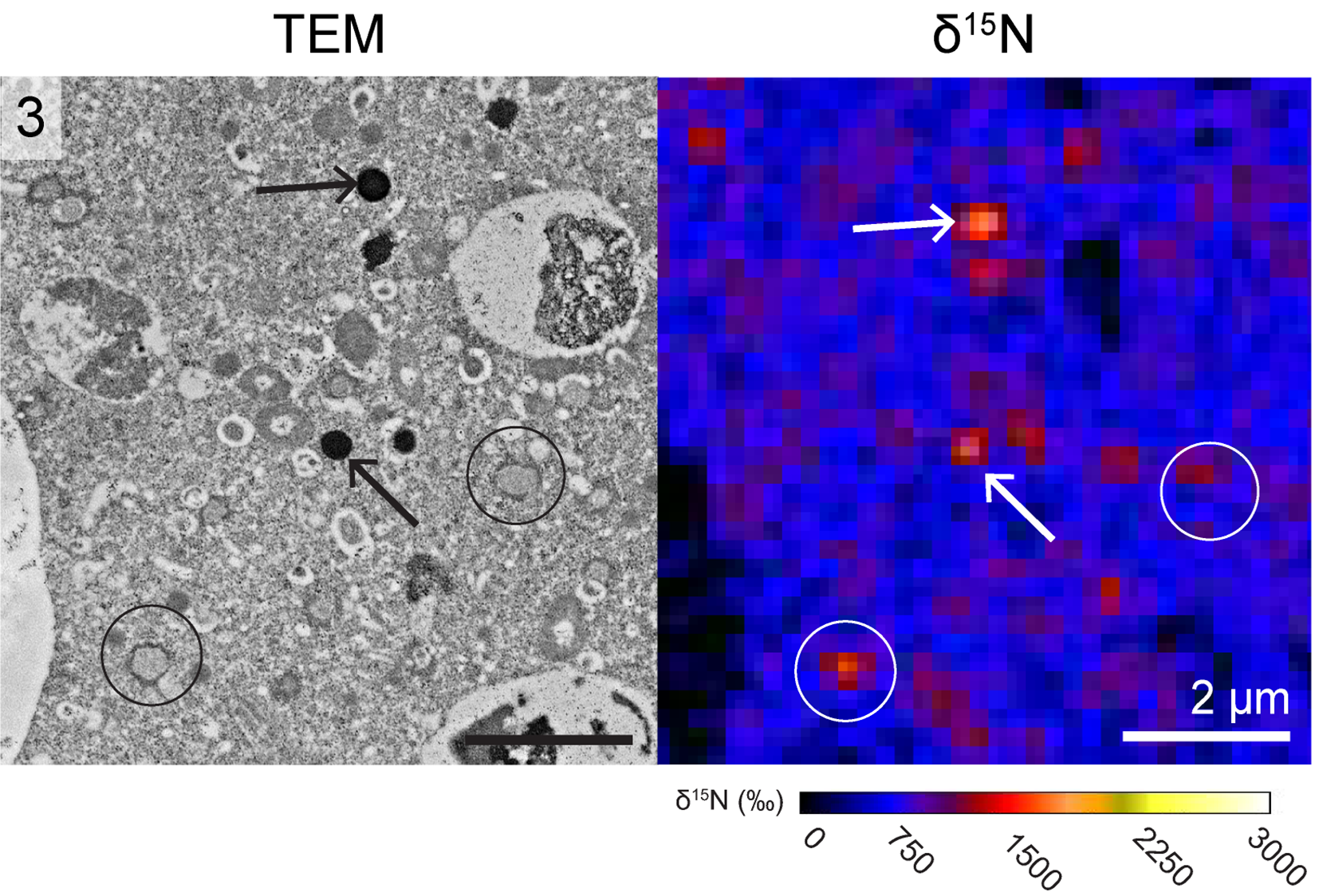

Supplement: Supplementary Figure 2 — 15N cellular localization in the electron-opaque bodies and fibrillar vesicles of G. bulloides after 6 h. Arrows: electron-opaque bodies; circles: fibrillar vesicles. [file Image_2.TIF]
