# Supplementary material for: Heterotrophic Foraminifera Capable of Inorganic Nitrogen Assimilation
Source: Front Microbiol. 2020 Dec 3;11:604979. doi: 10.3389/fmicb.2020.604979 (PMC7744380; doi:10.3389/fmicb.2020.604979)
Supplement: Supplementary Table 1 — Sampling information and experimental manipulations for each foraminiferal specimen collected. [file Table_1.DOCX]

| Morphospecies | Sample ID | ^13^C/^15^N exposure time (hours) | Sampling site | Sampling date | Co-ordinates | SST °C |
| --- | --- | --- | --- | --- | --- | --- |
| *Ammonia sp.* | 1 | 20 | Fiskebäckskil Harbor | 9 May 2016 | 58.241 N, 11.461 E | n/a |
| *Ammonia sp.* | 2 | 20 | Fiskebäckskil Harbor | 9 May 2016 | 58.241 N, 11.461 E | n/a |
| *Ammonia sp.* | 3 | 20 | Fiskebäckskil Harbor | 9 May 2016 | 58.241 N, 11.461 E | n/a |
| *Ammonia sp.* | 4 | 20 | Fiskebäckskil Harbor | 9 May 2016 | 58.241 N, 11.461 E | n/a |
| *Ammonia sp.* | 5 | 20 | Fiskebäckskil Harbor | 9 May 2016 | 58.241 N, 11.461 E | n/a |
| *Ammonia sp.* | 6 | 0 | Fiskebäckskil Harbor | 9 May 2016 | 58.241 N, 11.461 E | n/a |
| *Ammonia sp.* | 7 | 0 | Fiskebäckskil Harbor | 9 May 2016 | 58.241 N, 11.461 E | n/a |
| *Ammonia sp.* | 8 | 0 | Fiskebäckskil Harbor | 9 May 2016 | 58.241 N, 11.461 E | n/a |
| *Ammonia sp.* | 9 | 0 | Fiskebäckskil Harbor | 9 May 2016 | 58.241 N, 11.461 E | n/a |
| *Ammonia sp.* | 10 | 0 | Fiskebäckskil Harbor | 9 May 2016 | 58.241 N, 11.461 E | n/a |
| *Orbulina universa* | CONTROL1 | 0 | San Pedro Basin | Aug/Sept 2015 | 33.473 N, 118.485 W | 23.5 |
| *Orbulina universa* | CONTROL2 | 0 | San Pedro Basin | Aug/Sept 2015 | 33.473 N, 118.485 W | 23.5 |
| *Globigerina bulloides* | BUL1 | 6 | San Pedro Basin | Aug/Sept 2015 | 33.473 N, 118.485 W | 23.5 |
| *Globigerina bulloides* | BUL5 | 6 | San Pedro Basin | Aug/Sept 2015 | 33.473 N, 118.485 W | 23.5 |
| *Globigerina bulloides* | BUL3 | 18 | San Pedro Basin | Aug/Sept 2015 | 33.473 N, 118.485 W | 23.5 |
| *Globigerina bulloides* | BUL6 | 18 | San Pedro Basin | Aug/Sept 2015 | 33.473 N, 118.485 W | 23.5 |
